# Years of life lost to COVID-19 in 81 countries

**DOI:** 10.1038/s41598-021-83040-3

**Published:** 2021-02-18

**Authors:** Héctor Pifarré i Arolas, Enrique Acosta, Guillem López-Casasnovas, Adeline Lo, Catia Nicodemo, Tim Riffe, Mikko Myrskylä

**Affiliations:** 1 Centre for Research in Health Economics, Universitat Pompeu Fabra, 08002 Barcelona, Spain; 2 Max Planck Institute for Demographic Research, 18057 Rostock, Germany; 3 Department of Political Science, University of Wisconsin-Madison, 53706 Madison, WI USA; 4 Nuffield Department of Primary Care Health Science, University of Oxford, OX2 6GG Oxford, UK; 5 Center for Social Data Science, University of Helsinki, 00014 Helsinki, Finland

**Keywords:** Infectious diseases, Public health, Epidemiology

## Abstract

Understanding the mortality impact of COVID-19 requires not only counting the dead, but analyzing how premature the deaths are. We calculate years of life lost (YLL) across 81 countries due to COVID-19 attributable deaths, and also conduct an analysis based on estimated excess deaths. We find that over 20.5 million years of life have been lost to COVID-19 globally. As of January 6, 2021, YLL in heavily affected countries are 2–9 times the average seasonal influenza; three quarters of the YLL result from deaths in ages below 75 and almost a third from deaths below 55; and men have lost 45% more life years than women. The results confirm the large mortality impact of COVID-19 among the elderly. They also call for heightened awareness in devising policies that protect vulnerable demographics losing the largest number of life-years.

## Introduction

The large direct and indirect effects of the COVID-19 pandemic have necessitated the delivery of policy responses that, when reasonable, are a balancing act between minimizing the immediate health impact of the pandemic, and containing the long-term damage to the society that may arise from the protective policies. A key input parameter in the calculation of how restrictive policies might be justified is the mortality impact of COVID-19.

Attempts to evaluate the total mortality impact of COVID-19 are proceeding on several fronts. Progress is being made in estimating the infection fatality rate of COVID-19 and how this might vary across sub-populations^1^. Large, coordinated international collaborations have been set up to collect data that records COVID-19 attributable deaths. Attempts to estimate total excess mortality related to the COVID-19 are underway, and emphasized as an important measure^2,3^. Each of these research avenues and their associated health measures (infection rate, deaths and excess deaths) is important in informing the public and policymakers about the mortality impact of COVID-19. However, each come with their own limitations. Infection fatality rates apply only to the relatively small sub-population that has been confirmed to have the disease, and without knowledge about the true number of infected, these rates are inherently difficult to estimate. COVID-19 attributable deaths may over- or underestimate the true number of deaths that are due to the disease, as both policies and practices about coding the deaths are only being developed and standardized. Excess death approaches that compare mortality rates during the COVID-19 outbreak to a baseline depend on correctly estimating the baseline.

The most important limitation in COVID-19 attributable death or excess death approaches, however, is that these approaches do not provide information on how many life years have been lost. Deaths at very old ages can be considered to result in fewer life years lost, when compared to deaths at very young ages. In fact, several policy responses (or non-responses) have been motivated with the argument that COVID-19 is mostly killing individuals who, even in the absence of COVID-19, would have had few life years remaining. However, comprehensive evaluation of the true mortality impact of COVID-19 has not been conducted.

We analyze the premature mortality impact of COVID-19 by calculating the amount of life-years lost across 81 countries covering over 1,279,866 deaths. We base our analysis on two large recently established and continuously growing databases^4,5^ and on two different methodological approaches, one based on COVID-19 attributable deaths, and, for selected countries, one based on estimated excess deaths comparing recent mortality levels to an estimated baseline. We are not able to solve the measurement limitations of either of these approaches, but the complementary nature of the two ways of measuring COVID-19 deaths makes these concerns explicit and allows us to evaluate the implications. This study is also limited to premature mortality only; a full health impact evaluation might consider for instance, the burden of disability associated with the disease^6^. This latter dimension requires thorough understanding of sequelae associated with COVID-19, for which data are limited at this point on a cross-country, comparable level. As such, we focus on premature mortality here.

## Methods

Country death counts by age and gender due to COVID-19 come from the COVerAge-DB^4^; the analysis includes all countries with at least one COVID-19 related death in^4^ at the time of the study. Population data are drawn from the Human Mortality Database^5^ and the World Population Prospects^7^. Country life expectancies are from the life tables in the World Population Prospects for the period 2015-2020.

The dates at which data are collected, and death counts by country are reported in the Supplementary information materials (SI Table S1). Projections for total number of deaths due to COVID-19 by country are from the Imperial College^8^. Death counts due to other causes of mortality are from data in Global Burden of Disease^9^. Finally, we use weekly excess mortality data from the Short-Term Mortality Fluctuations Database (STMF, from the Human Mortality Database^5^). A full description of the data, its sources, and the methodology is provided in the Supplementary Information.

## Results

In total, 20,507,518 years of life have been lost to COVID-19 among the studied 81 countries, due to 1,279,866 deaths from the disease. The average years of life lost per death is 16 years. As countries are at different stages of the pandemic trajectory, this study is a snapshot of the impacts of COVID-19 on years of life lost (YLL) as of January 6, 2021 (a complete list of countries and their dates at measurement is in the Supplementary Information). In 35 of the countries in our sample, coverage of the data spans at least 9 months; in such cases, this suggests that the full impacts of the pandemic in 2020, or at least the first waves of the pandemic, are likely captured. For other countries still on an upwards incline of transmission rates or for which data is yet forthcoming for end of 2020, the YLL experienced are likely to further increase substantially in the next few months. We encourage context-based interpretation of the results presented here, especially when used for evaluation of the effectiveness of COVID-19 oriented policies.

**Figure 1:**
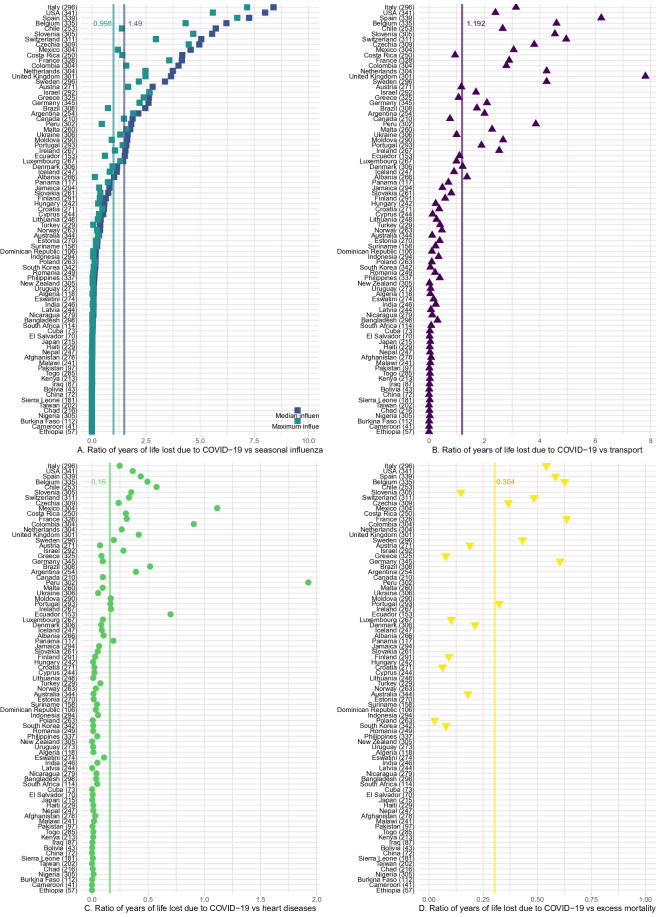
Panels A through C report the ratio of COVID-19 YLL rates over influenza YLL rates (in median/maximum deadly years by country), traffic accidents, and heart conditions respectively. Panel D reports, countries with available data, the ratio of YLL rates of COVID-19 deaths over YLL rates of excess deaths. When two causes of mortality affect YLL equally, the ratio is precisely 1; larger ratio values suggest COVID-19 YLL rates are higher than the alternative cause. Average ratios in vertical lines in each panel. Each country name is followed by (in parentheses) the number of days passed since the country’s first official COVID-case up to the last day of available COVID-19 deaths data for that country. Countries always sorted by ratio of COVID-19 YLL vs seasonal flu (in median years) across panels for ease of reading.

### Comparisons with other causes of mortality

To put the impacts of COVID-19 on YLL in perspective, we compare it against the premature mortality impacts of three other global common causes of death: heart conditions (cardiovascular diseases), traffic accidents (transport injuries), and the seasonal “flu” or influenza (see the Supplementary Information for definitions and cause ids). Heart conditions are one of the leading causes of YLL^6^, while traffic accidents are a mid level cause of YLL, providing sensible medium and high cause comparison baselines. Finally, common seasonal influenza has been compared against COVID-19, as both are infectious respiratory diseases (though see^10^, which suggests vascular aspects to the disease). We compare YLL rates (per 100,000) for COVID-19 against YLL rates for other causes of death. There is substantial variation in the mortality burden of seasonal influenza by country across years and so we compare YLL rates for the worst and median influenza years for each country in the period 1990–2017. Comparisons of YLL rates for COVID-19 over YLL rates for other causes are presented in Fig. 1.

We find that in heavily impacted highly developed countries, COVID-19 is 2–9 times that of the common seasonal influenza (as compared to a median flu year for the same country), between 2 and 8 times traffic related YLL rates, between a quarter and a half of the YLL rates attributable to heart conditions in countries (with rates as high as parity to twice that of heart conditions in Latin America). Variation across countries is large, as many countries have YLL rates due to COVID-19 still at very low levels. Results in our Supplementary Information show that these countries are often countries where relatively fewer days have passed since first confirmed case of COVID-19.

A noted problem in attributing deaths to COVID-19 has been systematic undercounting of deaths due to COVID-19, as official death counts may reflect limitations in testing as well as difficulties in counting in out-of-hospital contexts. In order to asses the importance of undercounting in our results, we compute excess deaths for 19 countries with available weekly mortality data. A mortality baseline is estimated for each country and age group for weekly all-cause mortality since the first week of 2010. Our results (Fig. 1, fourth panel) support the claim that the true mortality burden of COVID-19 is likely to be substantially higher. Comparisons of COVID-19 attributable deaths and excess deaths approaches to calculating YLL suggests that the former on average may underestimate YLL by a factor of 3. Variation across countries is large, in Belgium the two approaches deliver comparable results, but for Croatia, Greece and South Korea the excess deaths approach suggests that we may underestimate the YLL by a factor of more than 12.

**Figure 2:**
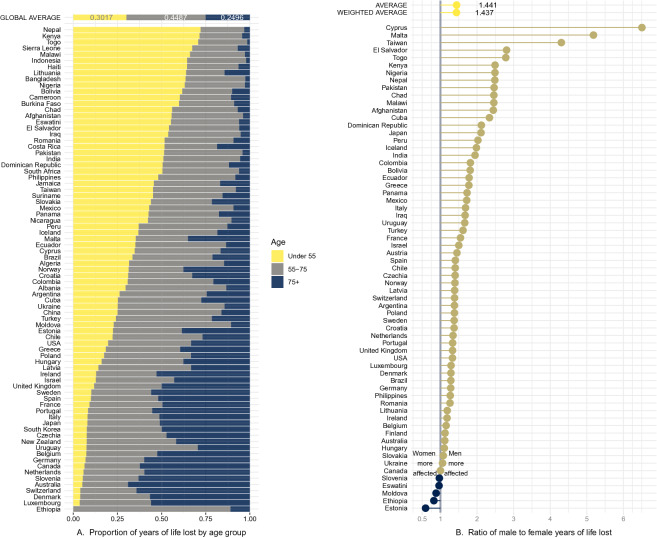
Panel A displays the country-specific proportions of YLL traced back to each age group. The global average proportion is presented at the top, and countries are in decreasing proportion of YLL in the under 55 age bracket. Panel B reports the ratio of male YLL rates to female YLL rates for countries with available gender specific COVID-19 death counts. Countries with genders equally affected by YLL rate are closer to the parity line at 1, while countries with women more affected have points lying on the left; countries with men more severely affected display points lying to the right. Global average and global weighted average of male to female YLL are presented at the top.

### Age specific years of life lost

As has been noted early on in the pandemic, mortality rates for COVID-19 are higher for the elderly^11^, with postulations that this may be due to correlations with the greater likelihood of these individuals suffering from underlying risk factors^12,13^. This study’s sample presents an average age-at-death of 72.9 years; yet only a fraction of the YLL can be attributed to the individuals in the oldest age brackets. Globally, 44.9% of the total YLL can be attributed to the deaths of individuals between 55 and 75 years old, 30.2% to younger than 55, and 25% to those older than 75. That is, the average figure of 16 YLL includes the years lost from individuals close to the end of their expected lives, but the majority of those years are from individuals with significant remaining life expectancy. Across countries, a substantial proportion of YLL can be traced back to the 55–75 age interval, however there remain stark differences in the relative contribution of the oldest and youngest age groups (Fig. 2, Panel A). These patterns account for the proportion of YLL for each age group out of the global YLL (see Table S7). In higher income countries, a larger proportion of the YLL is borne by the oldest group compared to the youngest age groups. The opposite pattern appears in low and mid-income countries, where a large fraction of the YLL are from individuals dying at ages 55 or younger.

### Gender specific years of life lost

It has also become apparent that there are gender disparities in the experience of COVID-19^14^; our study finds this to be true not only in mortality rates, but in absolute years of life lost as well. In the sample of countries for which death counts by gender are available, men have lost 44% more years than women. Two causes directly affect this disparity: (1) a higher average age-at-death of female COVID-19 deaths (71.3 for males, 75.9 for females), resulting in a relatively lower YLL per death (15.7 and 15.1 for males and females respectively); and (2) more male deaths than female deaths in absolute number (1.39 ratio of male to female deaths).

Though this general pattern is shared by most countries, the size of the disparity varies, as well as the importance of the two above causes. The ratio of male YLL rates (per 100,000) to female YLL rates for COVID-19 spans from near parity, such as in Finland or Canada, to more than double the YLL rates countries like Peru or quadruple like in Taiwan (Fig. 2, Panel B). For countries that present highly skewed male to female YLL rates (most prevalent in low-income countries), the death count differences across genders contribute the most to this imbalance. Yet, the substantial imbalances remain starkly present among high-income countries as well (see Supplementary Information for details).

## Discussion

Understanding the full health impact of the COVID-19 pandemic is critical for evaluating the potential policy responses. We analyzed the mortality impact of COVID-19 by calculating the amount of life-years lost across 81 countries covering over 1,279,866 deaths. From a public health standpoint, years of life lost is crucial in that it assesses how much life has been cut short for populations affected by the disease. We considered COVID-19 attributable deaths throughout in identifying patterns of years of life lost, and as an important robustness check, conducted analysis based on estimated excess deaths comparing recent mortality levels to a (estimated) baseline. Our results deliver three key insights. First, the total years life lost (YLL) as of January 06, 2021 is 20,507,518, which in heavily affected countries is between 2 and 9 times the median YLL of seasonal influenza or between a quarter and a half of heart disease. This implies 273,947 “full lives lost” – or over two hundred thousand lives lived from birth to the average life expectancy at birth in our sample (74.85 years). Second, three quarters of the YLL are borne by people dying in ages below 75. Third, men have lost 45% more years of life than women.

These results must be understood in the context of an as-of-yet ongoing pandemic and after the implementation of unprecedented policy measures. Existing estimates on the counterfactual of no policy response suggest much higher death tolls and, consequently, YLL. Our calculations based on the projections by^8^ yield a total impact several orders of magnitude higher, especially considering projections based on a complete absence of interventions (see Supplementary Information for details on projections). This is in line with further evidence of the life-saving impacts of lockdowns and social distancing measures^15^.

There are two key sources of potential bias to our results, and these biases operate in different directions. First, COVID-19 deaths may not be accurately recorded, and most of the evidence suggests that on the aggregate level, they may be an undercount of the total death toll. As a result, our YLL estimates may be underestimates as well. We compare our YLL estimates to estimates based on excess death approaches that require more modeling assumptions but are robust to missclassification of deaths. The results of this comparison suggest that on average across countries, we might underestimate COVID-19 YLL rates by a factor of 3.

Second, those dying from COVID-19 may be an at-risk population whose remaining life expectancy is shorter than the average person’s remaining life expectancy^16–18^. This methodological concern is likely to be valid, and consequently our estimate of the total YLL due to COVID-19 may be an overestimate. However, our key results are not the total YLL but YLL ratios and YLL distributions which are relatively robust to the co-morbidity bias. Indeed, this bias also applies to the YLL calculations for the seasonal influenza or heart disease. Thus, the ratio of YLL for COVID-19 compared to other causes of death is more robust to the co-morbidity bias than the estimate on the level of YLL as the biases are present in both the numerator and the denominator. Likewise, the age- and gender distributions of YLL would suffer from serious co-morbidity bias only if these factors vary strongly across the age or gender spectrum.

As noted earlier, our analysis is limited to premature mortality. A full health impact evaluation ought to consider the burden of disability associated with the disease.Indeed, YLL are often presented jointly with years lived with disability (YLD) in a measure known as disability-adjusted life year (DALY), constructed by adding YLD to YLL^19^. In order to compute YLD, though, we must have a thorough understanding of the sequela associated with the disease, as well their prevalence. Several sequelae have been linked to COVID-19 recently^20,21^ in China, but we still lack the full understanding of the extent that would be needed to compute reliable cross-national YLD measures at the scale of this article. We see collection of such measures as therefore of key importance in next steps in advancing our understanding of the magnitude of the COVID-19 effects on public health.

Some of our findings are consistent with dominant narratives of the COVID-19 impact, others suggest places where more nuanced policy-making can affect how the effects of COVID-19 might be spread among society. Our results confirm that the mortality impact of COVID-19 is large, not only in terms of numbers of death, but also in terms of years of life lost. While the majority of deaths are occurring at ages above 75, justifying policy responses aimed at protecting these vulnerable ages, our results on the age pattern call for heightened awareness of devising policies protecting also the young. The gender differential in years of life lost arises from two components: more men are dying from COVID-19, but men are also dying at younger ages with more potential life years lost than women. Holding the current age distribution of deaths constant, eliminating the gender differential in YLL would require on average a 34% reduction in male death counts; this suggests that gender-specific policies might be equally well justified as those based on age.

## Supplementary Information

Supplementary Information

## Data Availability

All study code and data are fully replicable and available in the following Open Science Framework (OSF) repository: https://osf.io/5j9nc/?view_only=48f0f69952814e3a8e967370e7b50954.
