# Author Correction: Years of life lost to COVID-19 in 81 countries

**DOI:** 10.1038/s41598-021-87640-x

**Published:** 2021-04-14

**Authors:** Héctor Pifarré i Arolas, Enrique Acosta, Guillem López‑Casasnovas, Adeline Lo, Catia Nicodemo, Tim Riffe, Mikko Myrskylä

**Affiliations:** 1 Centre for Research in Health Economics, Universitat Pompeu Fabra, 08002 Barcelona, Spain; 2 Max Planck Institute for Demographic Research, 18057 Rostock, Germany; 3 Department of Political Science, University of Wisconsin-Madison, Madison, WI 53706 USA; 4 Nuffield Department of Primary Care Health Science, University of Oxford, Oxford, OX2 6GG UK; 5 Center for Social Data Science, University of Helsinki, 00014 Helsinki, Finland

Correction to: *Scientific Reports* 10.1038/s41598-021-83040-3, Published on 18 February 2021

The Supplementary Information published with this Article contained an error, where Table S5 was a duplicate of Table S1.

As a result in the Supplementary Information, in the ‘This PDF file includes’ section and Methods section under the subheading of ‘Calculation of years of life lost (YLL)’ the in text references to Table S1 was incorrectly given as Table S5.

This error has now been corrected in the Supplementary Information file that accompanies the original Article. The corrected Supplementary Information file is also linked to this correction notice.

## Supplementary Information

Supplementary Information

