# Supplementary material for: Years of life lost to COVID-19 in 81 countries
Source: Sci Rep. 2021 Feb 18;11:3504. doi: 10.1038/s41598-021-83040-3 (PMC7892867; doi:10.1038/s41598-021-83040-3)
Supplement: Supplementary file 1 — Supplementary Information [file 41598_2021_83040_MOESM1_ESM.pdf]

Supplementary Information:
Years of life lost to COVID-19 in 81 countries

Héctor Pifarré i Arolas,[1*] Enrique Acosta,[2] Guillem López Casasnovas[1],
Adeline Lo[3], Catia Nicodemo[4], Tim Riffe [2], & Mikko Myrskylä[2,5]

[1]Centre for Research in Health Economics, Universitat Pompeu Fabra, 08002 Barcelona, Spain
[2]Max Planck Institute for Demographic Research, 18057 Rostock, Germany
[3]Department of Political Science, University of Wisconsin-Madison, 53706 Madison, WI
[4]Centre of Organisation, Department of Primary Economics, University of Oxford, OX1 3UQ Oxford, UK
[5]Center for Social Data Science, University of Helsinki, 00014 Helsinki, Finland

[*]To whom correspondence should be addressed; E-mail: hector.pifarre@upf.edu.

March 31, 2021

This PDF file includes:

- Methods: a description of the methods and data sources.
- Additional tables and figures:
  - Description of SI Figures and Tables
  - Figures S1-S11
  - Tables S1-S7

Methods

Calculation of years of life lost (YLL)

To measure the impact of COVID-19 in terms of premature mortality, in the outbreak spanning January 2020 to December 2020[1], we calculate the years of life lost (YLL) for a person who dies at a given age due to COVID-19 as the difference between their age at death and their life expectancy at that given age [1].

The age specific life expectancy is extracted from the country-specific life tables in WPP. This approach differs slightly from existing YLL exercises such as the GBD study [2] which uses a single best-practices life table to measure life expectancy countries. Here country-specific tables are more applicable under the premise that the focus of the study is not of trends, but rather in evaluating the magnitude of the premature mortality impacts of COVID-19 at this time. For the same reason, we additionally compute YLL rates per 100,000 people based on country specific population, instead of the more common route of utilizing a standard population. In this setting, our YLL rates do not disentangle the effects of country-specific age structures

[1]Per country span of data differs, see Table S1 for details.

Comparisons with other causes of mortality

For heart conditions (cardiovascular diseases) and traffic accidents (transport injuries), we use mortality data collected from the GBD [3] in 2017 (cause IDs 491 and 688 respectively on the database). There is substantial variation in the mortality burden of the flu by country across years. To better illustrate the context, we calculate YLL rates for the worst flu and median flu years for each country in the period 1990-2017. We select years according to the YLL rates following our approach described above.

Selection of Respiratory infections and Pneumonia and Influenza as causes of death

The identification of influenza mortality is challenging because influenza tests are rarely performed, and substantial influenza-related mortality is the consequence of exacerbation of preexisting medical conditions. Therefore, most of the deaths from influenza are classified within other causes, such as respiratory and cardiovascular diseases. Usually, the estimation of influenza mortality is done by applying a Serfing model to monthly or weekly mortality to broader groups of causes of death, ideally including influenza surveillance information [4].

Nevertheless, for most of the countries under analysis, influenza epidemics severity had considerable differences and the detailed information for obtaining direct measures of mortality from influenza by age is not available. For comparability reasons, we selected all-cause mortality involving pneumococcal pneumonia, H influenzae type B, and influenza as etiology from the GDB data [3] (cause IDs 188, 189, and 187 respectively on the databases).

Excess mortality

We compute excess deaths for 19 countries with available weekly mortality data. A mortality baseline is estimated for each country, sex and age group by fitting a Poisson model to weekly all-cause mortality since the first week of 2010. Mortality excess here is defined as the difference between observed mortality and the baseline. For the calculation of excess mortality attributable to COVID-19, we compute the cumulative positive excess mortality since the last week of February 2020. This is then compared to official death counts for COVID-19 around comparable dates.

The model has three main components: a sinusoidal component accounting for annual seasonality, a nonlinear trend component using 3-knots splines accounting for secular changes in mortality, and an offset controlling for changes in the exposure over time. In each case, the inclusion of the seasonal component is decided by selecting the model with the best AIC value [5].

Mortality excess is defined as the observed mortality minus the baseline. We compute bootstrapped 95% prediction intervals of the excess mortality (2000 iterations). For the calculation of excess mortality attributable to COVID-19, we calculate the cumulative positive excess mortality since the last week of February 2020. Results are reported in SI Table S3. As noted by the HMD, the mortality counts during the most recent weeks (one to three) is incomplete due to delayed registration of deaths [6]. Thus, to ensure data completeness, we excluded the last three weeks in all countries for the estimation of excess mortality.

Projections

It is inherently difficult to extricate these measures from the policy contexts in which they are calculated. Are certain countries experiencing lower YLL than others due to more rigid policy measures (e.g. stay-at-home orders, mask enforcement)? To attain some sense of policy context, we take projections of the number of COVID-19 deaths in 2020 under a policy scenario of no intervention, with different disease transmission (so-called R_0) rates [7] and produce similar calculations of years of life lost. The data used is obtained from the original report behind the publication and is retrieved from: `https://www.imperial.ac.uk/media/imperial-college/medicine/sph/ide/gida-fellowships/Imperial-College-COVID19-Global-unmitigated-mitigated-suppression-scenarios.xlsx`. The results of these exercises are shown in SI Appendix Table S3.

Gender imbalances

SI Appendix Table S4 reports key figures behind the gender disparity in the impact of COVID-19, by country: 1) a higher average age-at-death of female COVID-19 deaths, resulting in a relatively lower YLL per death (16.6 and 16 for males and females respectively); and 2) more male deaths than female deaths in absolute number.

In order to provide a policy relevant magnitude of the gender imbalance, we calculate the reduction in male death counts needed for perfect parity. We hold differential age pattern of death across genders constant and reduce male death counts up to the point in which YLL rates are equal across genders. The resulting death counts reductions are presented in SI Appendix Table S5 in terms of the ratio of equalizing to current death counts. The mean across countries, weighted by number of deaths and unweighted, is 0.69 and 0.66 respectively.

Additional tables and figures

Sample description and additional information Detailed key information on the sample and on the calculations reported in the main text.

- Table S1 reports sample of countries including in the analysis and, for each country, the reference date, overall and gender specific death counts.
- Table S2 reports the sub-sample used in the excess mortality calculations, with its specific reference dates and overall cumulative actual death counts and estimated excess deaths.
- Table S3 compares figures of both death counts and YLL for the current and projected impact of COVID-19.
- Table S4 reports gender specific measures of the impact of the pandemic by country.
- Table S5 displays the reduction in male deaths needed to equalize the impact of the pandemic across genders in terms of YLL.
- Table S6 reports the weighted averages for Figure 1 in the main text, and Table S7 for those in Figure 2.

Pandemic stage and premature mortality impact Countries are experiencing different stages of the pandemic trajectory. We measure that stage based on the days since the first COVID-19 case and provide suggestive evidence that later stages are associated with a higher burden of premature mortality. Figures S1 through S6 investigate this plot this relationship for a variety of outcomes. Figures S7, S8 and S9 show alternative representations of the age and gender imbalance in YLL. Figures S10 and S11 provide additional information on the excess mortality estimates.

- Figure S1 plots the growth of years of life lost with increase of days from first COVID-19 case.
- Figure S2 plots the ratio the ratio COVID-19 YLL over other causes of mortality YLL and excess mortality, graphed against increase of days from first COVID-19 case.
- Figure S3 through S6 display the plots in figure S2 separately.
- Figure S7 plots the proportion of YLL from two age groups as days from first COVID-19 case increases.
- Figure S8 plots YLL rates from male and female subgroups as days from first COVID-19 case increases.
- Figure S9 plots replicates Figure 2 from the main manuscript, with ratio of male YLL rates over female YLL rates on log x-axis scale.

Excess mortality Supplementary information on the output of the predictive models of mortality.

- Figures S10 and S11 report the excess mortality and death counts estimates from our excess mortality models.

Figures S1 to S11

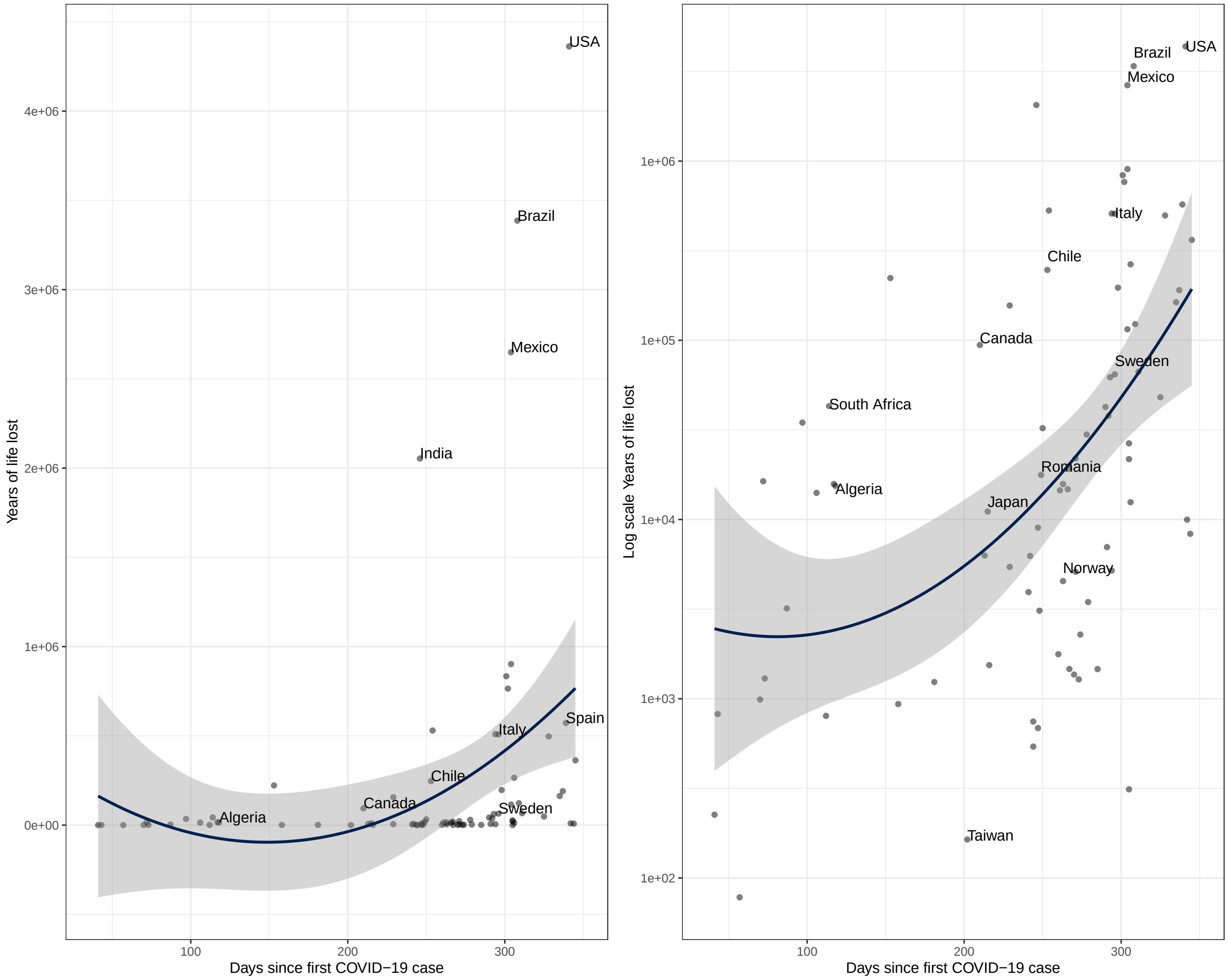

Figure S1: Growth of years of life lost with increase of days from first official COVID-19 case. Left panel plots days of first case against years of life lost, while right panel plots days of first case against log scale years or life lost. Polynomial order 2 curve fit against data.

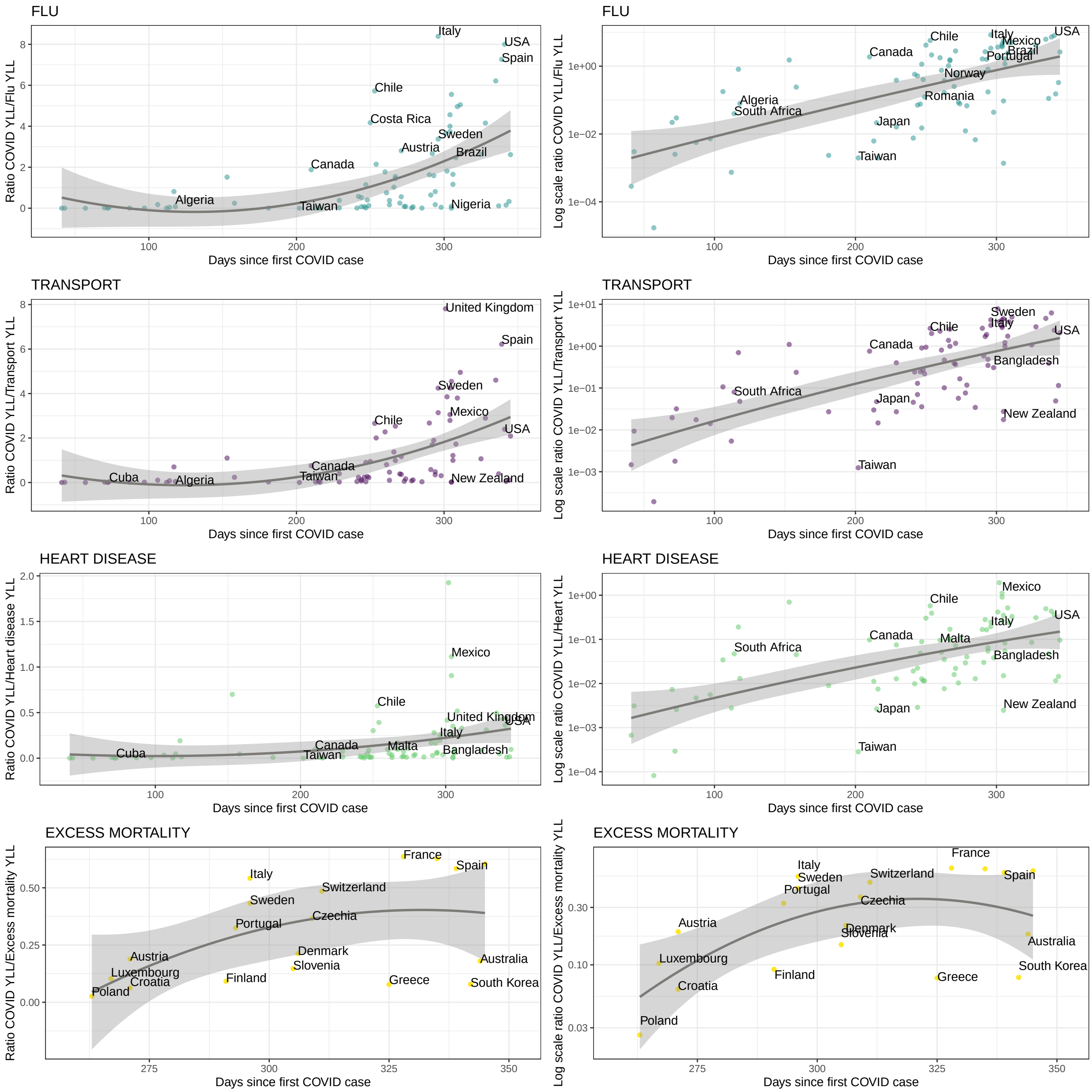

Figure S2: Ratio COVID-19 YLL over other causes of mortality YLL, graphed against increase of days from first COVID-19 case. Left column plots days of first case against ratio, while right panel plots days of first case against log scale ratio. Flu year used is median impact flu year for each country. Polynomial order 2 curve fit against data.

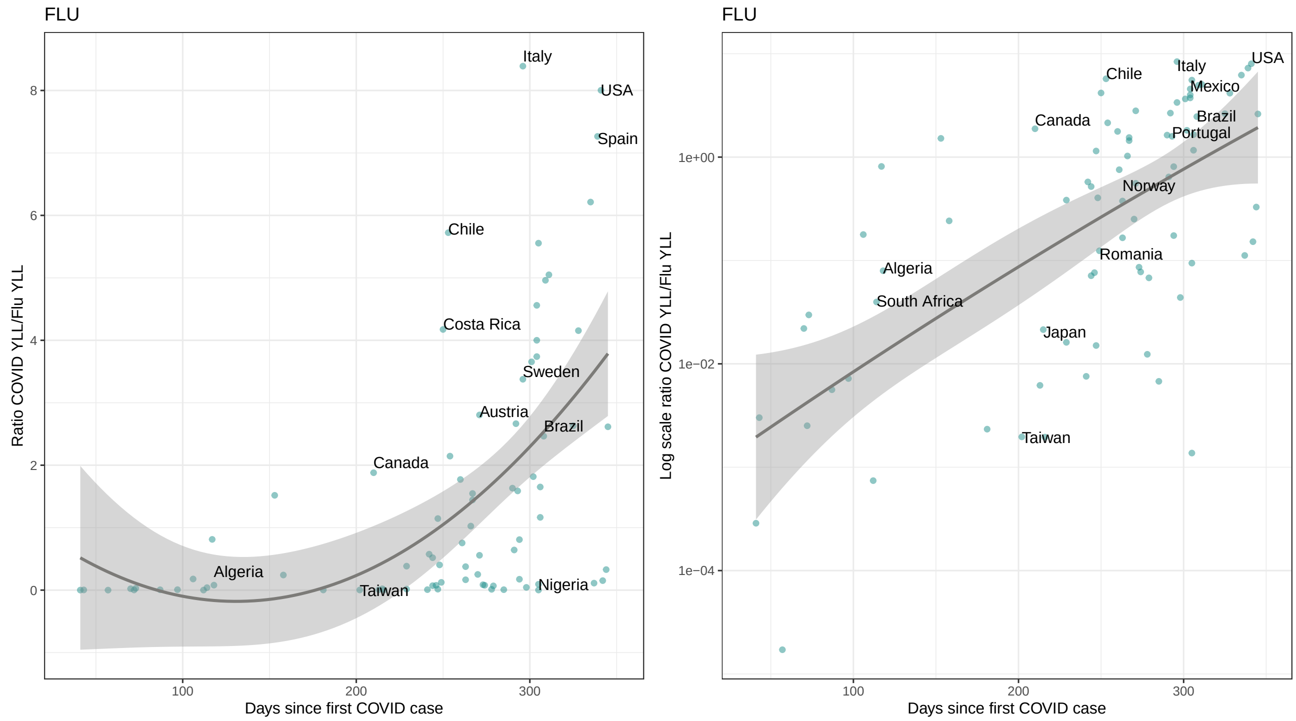

Figure S3: Ratio COVID-19 YLL over flu mortality YLL, graphed against increase of days from first COVID-19 case. Left plots days of first case against ratio, while right plots days of first case against log scale ratio. Flu year used is median impact flu year for each country. Polynomial order 2 curve fit against data.

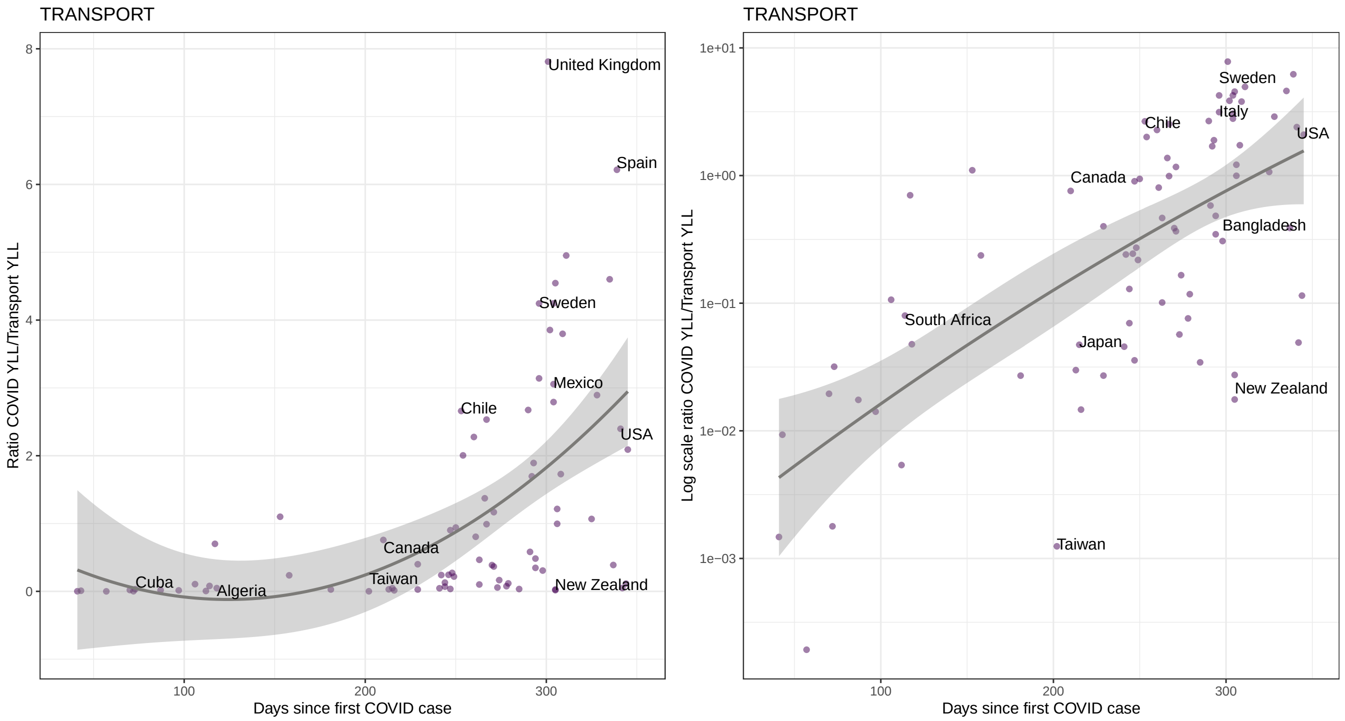

Figure S4: Ratio COVID-19 YLL over transport mortality YLL, graphed against increase of days from first COVID-19 case. Left plots days of first case against ratio, while right plots days of first case against log scale ratio. Polynomial order 2 curve fit against data.

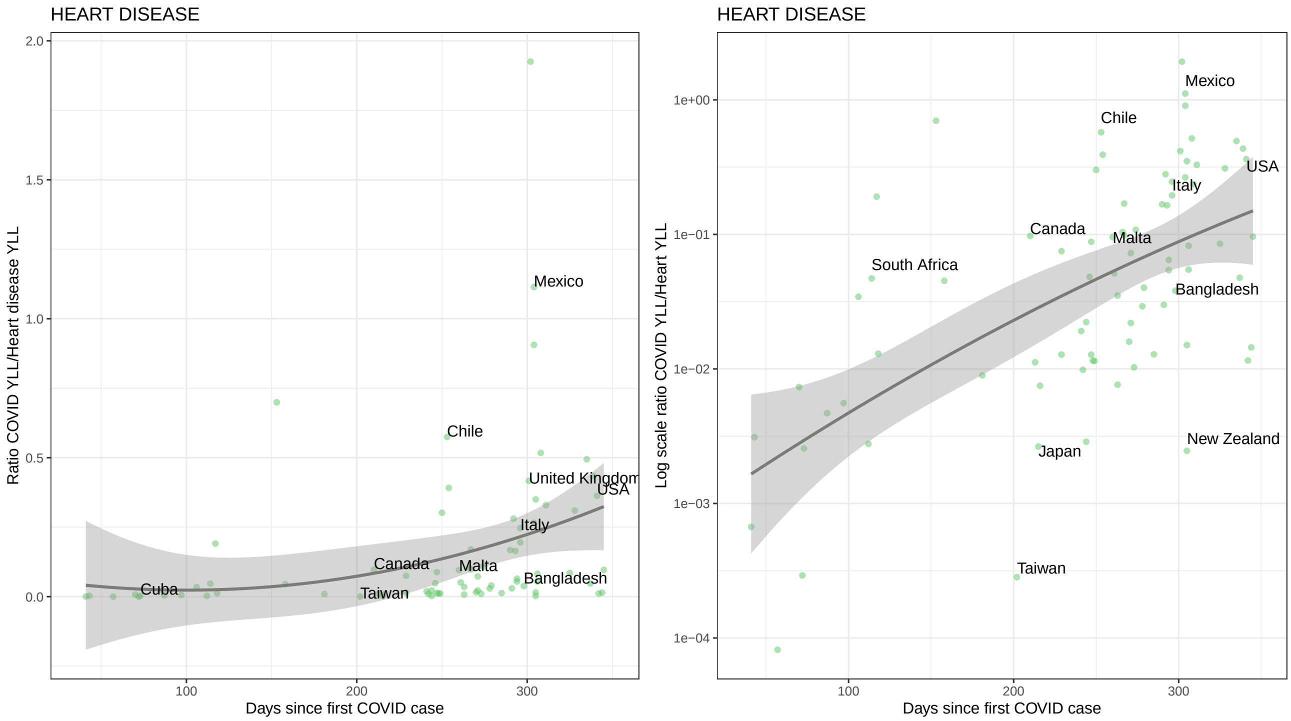

Figure S5: Ratio COVID-19 YLL over heart disease YLL, graphed against increase of days from first COVID-19 case. Left plots days of first case against ratio, while right plots days of first case against log scale ratio. Polynomial order 2 curve fit against data.

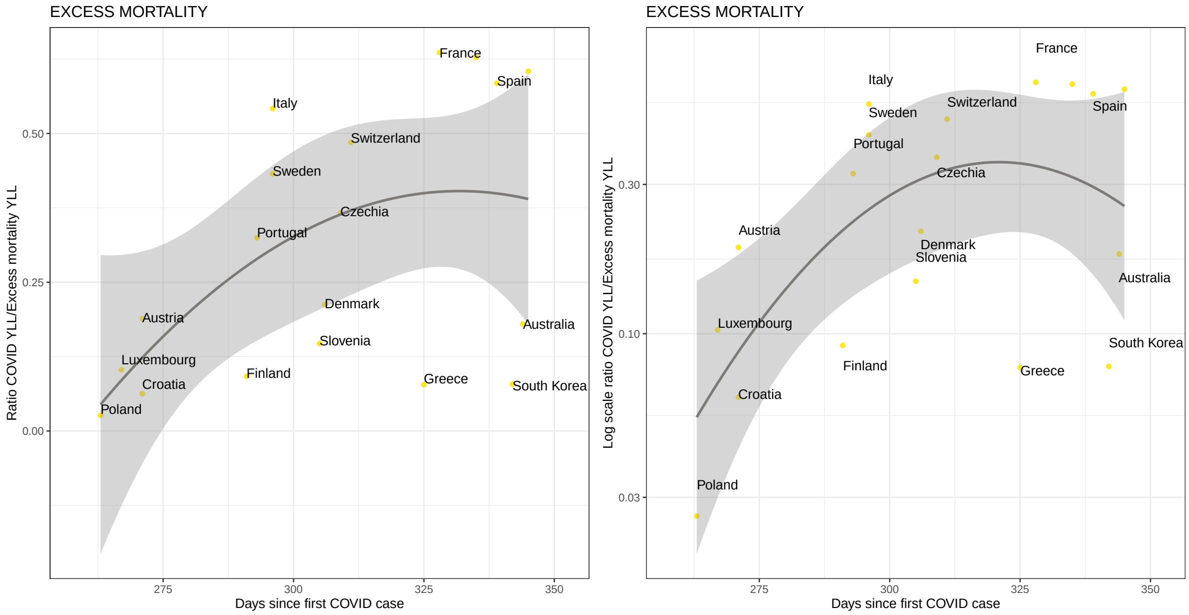

Figure S6: Ratio COVID-19 YLL over excess mortality YLL, graphed against increase of days from first COVID-19 case. Left plots days of first case against ratio, while right plots days of first case against log scale ratio. Polynomial order 2 curve fit against data.

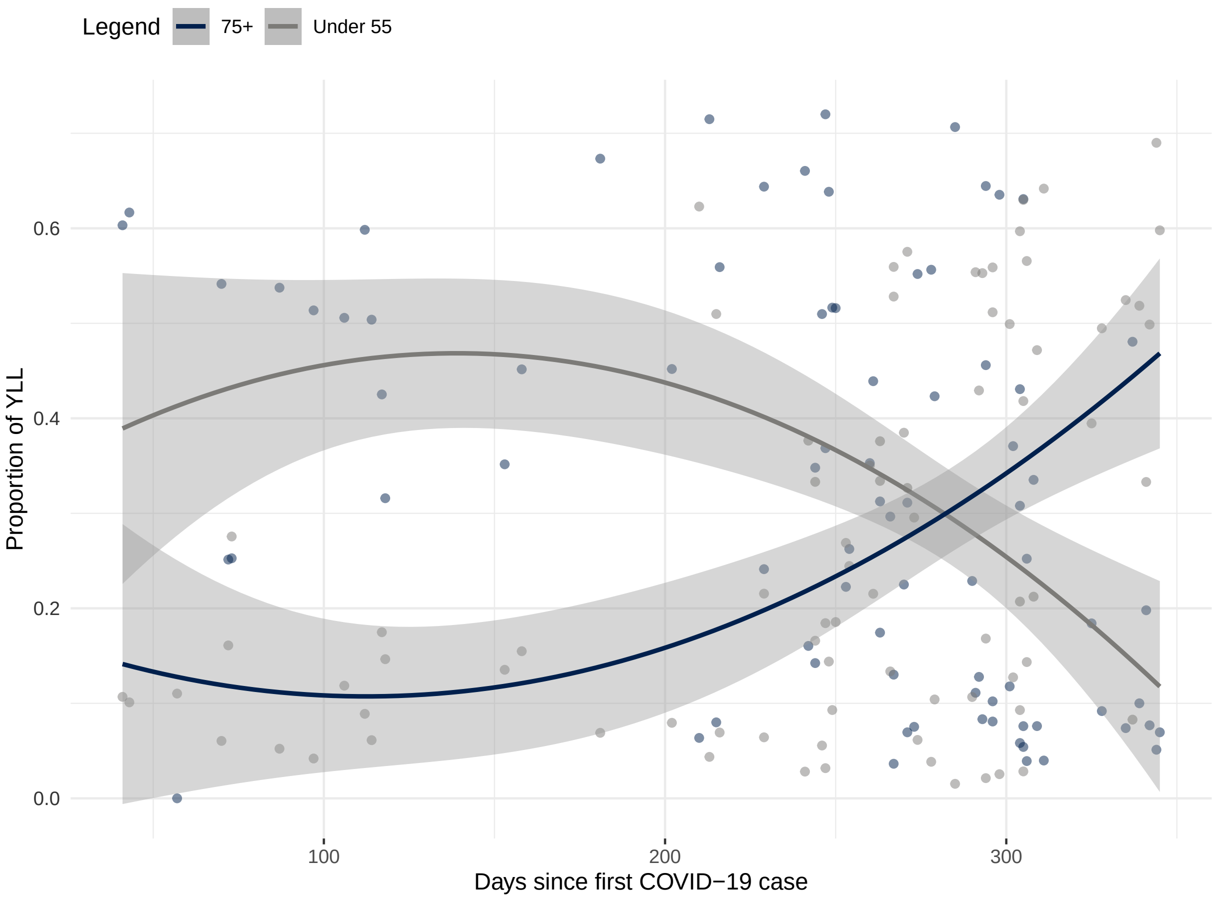

Figure S7: Proportion of YLL from two age groups as days from first COVID-19 case increases. Polynomial order 2 curve fit against data.

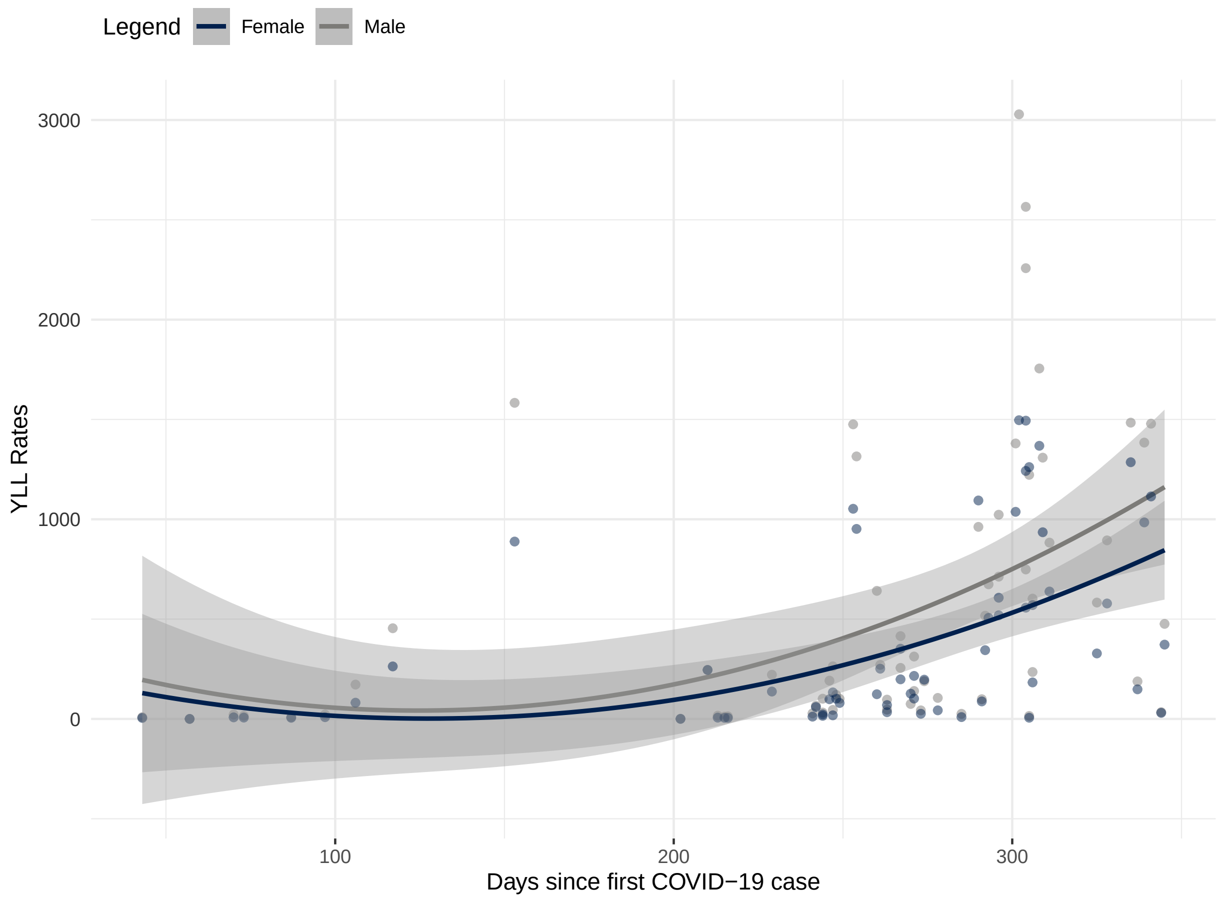

Figure S8: YLL rates from male and female subgroups as days from first COVID-19 case increases. Polynomial order 2 curve fit against data.

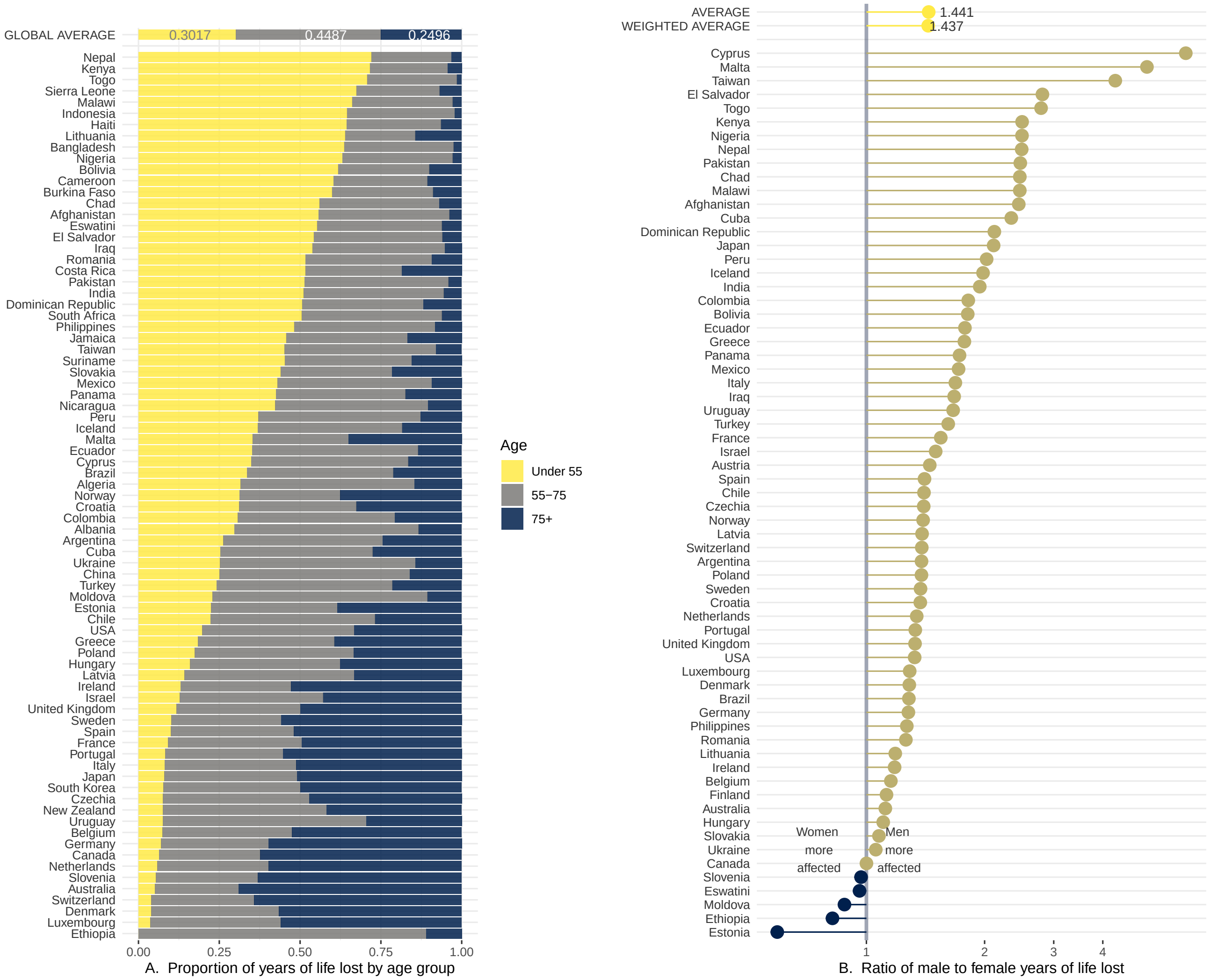

Figure S9: Replication of Figure 2 from main manuscript, with ratio of male YLL rates over female YLL rates on log x-axis scale.

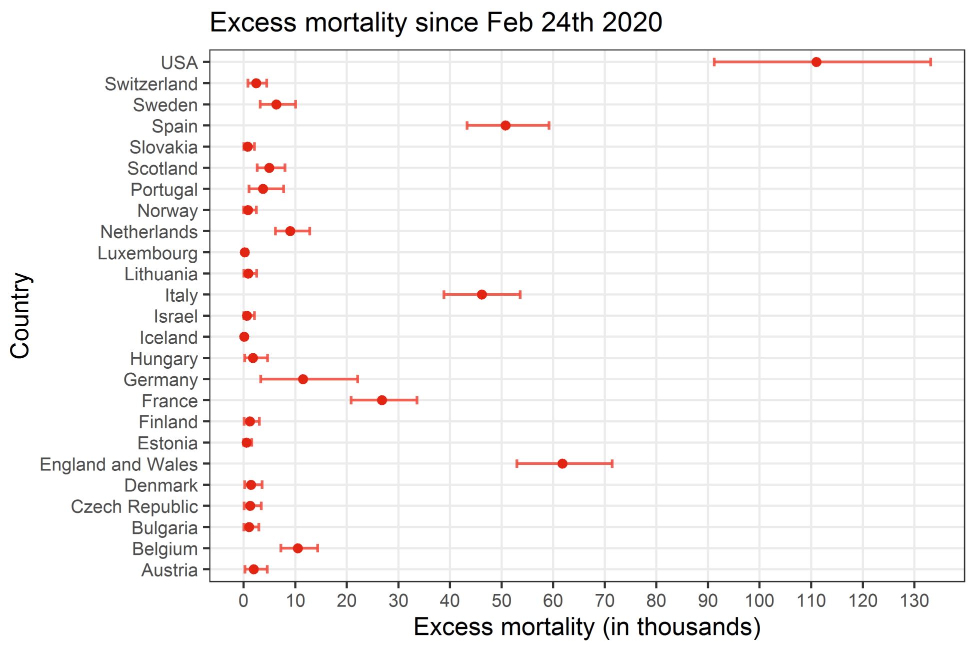

Figure S10: Excess mortality and its 95% prediction interval by country, for all ages and both sexes. Excess mortality is defined as the difference between the observed and predicted mortality baseline, between February 24, 2020 and the last week in which mortality was observed in each country.

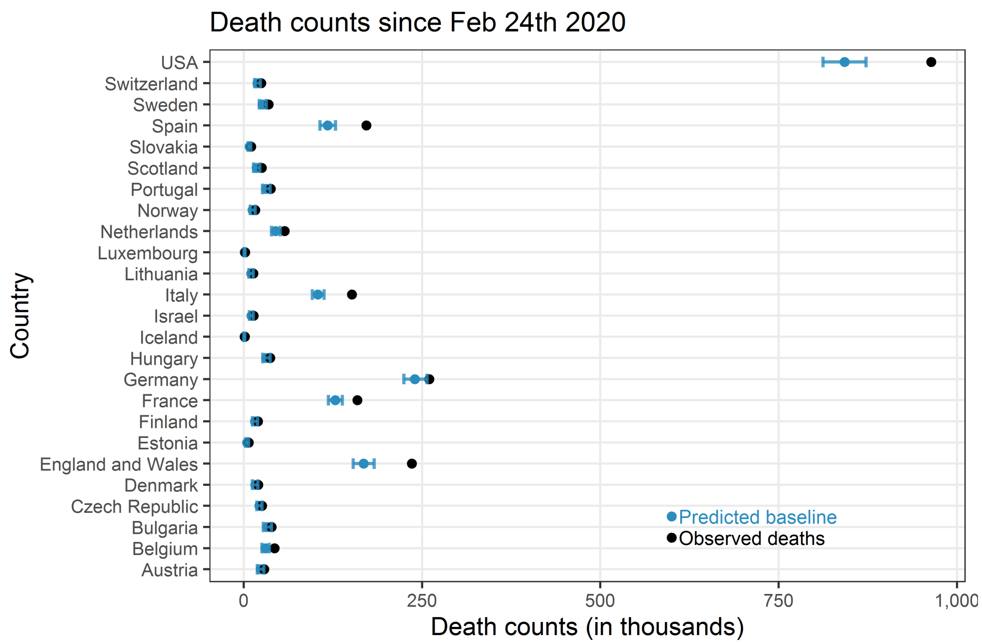

Figure S11: Total observed (in black) and predicted mortality baseline (in blue), for all ages and both sexes, between February 24, 2020 and the last week of observation in each country.

Tables S1 to S7

Table S1: COVID-19 attributed deaths by date, country and gender.

Country	Date	Both	Males	Females
Afghanistan	2020-11-28	1511	1130	381
Albania	2020-12-02	839	NA	NA
Algeria	2020-06-22	852	NA	NA
Argentina	2020-11-12	34759	19786	14973
Australia	2021-01-03	910	440	470
Austria	2020-11-22	2151	1366	1107
Bangladesh	2020-12-31	7559	NA	NA
Belgium	2021-01-04	19724	9652	10072
Bolivia	2020-04-22	34	22	12
Brazil	2020-12-29	186406	106654	79753
Burkina Faso	2020-06-30	53	NA	NA
Cameroon	2020-04-16	18	13	5
Canada	2020-09-22	9188	4435	4754
Chad	2020-10-21	92	70	22
Chile	2020-11-11	14777	8463	6314
China	2020-02-11	1023	NA	NA
Colombia	2021-01-04	49665	31736	17929
Costa Rica	2020-11-10	1502	NA	NA
Croatia	2020-11-22	400	208	192
Cuba	2020-05-24	82	52	30
Cyprus	2020-11-08	42	32	10
Czechia	2021-01-04	11258	6314	4944
Denmark	2020-12-29	1226	684	542
Dominican Republic	2020-06-15	615	433	182
Ecuador	2020-07-31	10615	7025	3590
El Salvador	2020-05-28	42	31	11
Estonia	2020-11-22	111	42	69
Eswatini	2020-12-10	126	72	54
Ethiopia	2020-05-09	5	2	3
Finland	2020-11-15	669	204	210
France	2020-12-17	41299	24268	17031
Germany	2021-01-04	35514	18573	16941
Greece	2020-12-16	3870	2314	1556
Haiti	2020-11-04	232	NA	NA
Hungary	2020-11-01	539	264	275
Iceland	2020-11-01	35	20	15
India	2020-10-02	100875	69887	30988
Indonesia	2020-12-21	20085	NA	NA
Iraq	2020-05-24	160	110	50
Ireland	2020-11-22	1758	914	844
Israel	2020-12-09	2932	1670	1262
Italy	2020-11-22	43793	25257	18536
Jamaica	2020-12-29	298	NA	NA
Japan	2020-08-18	825	531	294
Kenya	2020-10-11	280	210	70
Latvia	2020-11-01	42	23	19
Lithuania	2020-11-01	138	68	70
Luxembourg	2020-11-22	159	82	77

Table S1: COVID-19 attributed deaths by date, country and gender. *(continued)*

**Country**	**Date**	**Both**	**Males**	**Females**
Malawi	2020-11-29	185	141	44
Malta	2020-11-22	121	90	31
Mexico	2020-12-28	122855	77991	44864
Moldova	2020-12-23	2825	1457	1368
Nepal	2020-09-26	360	258	102
Netherlands	2020-12-27	11303	6181	5122
New Zealand	2020-12-29	25	NA	NA
Nicaragua	2020-12-23	164	NA	NA
Nigeria	2020-11-28	1163	869	294
Norway	2020-11-15	353	189	164
Pakistan	2020-06-02	1688	1252	436
Panama	2020-07-04	720	478	242
Peru	2021-01-02	37835	25885	11950
Philippines	2021-01-01	9213	5554	3659
Poland	2020-11-22	1132	630	502
Portugal	2020-12-20	6191	3220	2971
Romania	2020-11-01	842	500	342
Sierra Leone	2020-09-28	72	NA	NA
Slovakia	2020-11-22	878	452	426
Slovenia	2021-01-03	2891	1319	1572
South Africa	2020-06-27	2413	NA	NA
South Korea	2020-12-27	808	NA	NA
Spain	2021-01-04	50900	27838	23062
Suriname	2020-08-18	54	NA	NA
Sweden	2020-11-22	6553	3563	2990
Switzerland	2021-01-01	7082	3811	3272
Taiwan	2020-08-10	7	6	1
Togo	2020-12-16	66	45	21
Turkey	2020-10-26	9799	6042	3756
Ukraine	2021-01-03	17395	9298	8097
United Kingdom	2020-11-27	73124	40213	32911
Uruguay	2020-12-11	90	59	31
USA	2020-12-26	301671	163633	138038

Table S2: Estimated excess deaths and COVID-19 deaths.

**Country**	**Date**	**Excess deaths**	**COVID-19 deaths**
Australia	2020-10-04	2562	860
Austria	2020-11-08	5174	1300
Belgium	2020-11-29	23599	16748
Croatia	2020-11-08	4093	197
Czechia	2020-11-01	9487	3812
Denmark	2020-12-13	2751	950
Finland	2020-12-06	3168	415
France	2020-11-15	55628	30871
Germany	2020-11-22	24552	19523
Greece	2020-10-11	5364	449
Italy	2020-10-11	68154	36233
Luxembourg	2020-11-15	583	141
Poland	2020-11-22	50801	1132
Portugal	2020-11-29	12092	4505
Slovenia	2020-11-01	2036	440
South Korea	2020-09-13	3868	358
Spain	2020-11-29	85180	46438
Sweden	2020-11-08	9951	6016
Switzerland	2020-11-22	6417	3788

Table S3: Current and projected: COVID-19 deaths and YLL rates.

Country	Deaths Current	Projected	YLL rates Current	Projected	Ratio of YLL Current over proj.
Afghanistan	1511	74381	76.568	3768.934	49.223
Albania	839	15342	512.413	9367.771	18.282
Algeria	852	171534	35.136	7074.746	201.355
Argentina	34759	212412	1172.818	7167.122	6.111
Australia	910	146707	32.645	5262.867	161.216
Austria	2151	59573	243.525	6744.532	27.695
Bangladesh	7559	592125	119.341	9348.323	78.333
Belgium	19724	91586	1420.122	6594.200	4.643
Bolivia	34	41442	7.042	8557.712	1215.308
Brazil	186406	908009	1593.215	7760.778	4.871
Burkina Faso	53	36433	3.840	2639.983	687.415
Cameroon	18	50535	0.849	2411.725	2839.045
Canada	9188	266741	249.430	7241.392	29.032
Chad	92	28507	9.376	2908.397	310.196
Chile	14777	96205	1291.462	8408.005	6.510
China	1023	8642939	1.136	9595.971	8447.795
Colombia	49665	208179	1772.999	7431.816	4.192
Costa Rica	1502	22929	634.897	9692.755	15.267
Croatia	400	28100	124.519	8749.644	70.268
Cuba	82	69278	11.460	9681.790	844.854
Cyprus	42	6392	61.774	9401.475	152.190
Czechia	11258	67843	1156.169	6967.308	6.026
Denmark	1226	37521	214.595	6567.011	30.602
Dominican Republic	615	39710	129.872	8385.740	64.569
Ecuador	10615	63996	1260.959	7602.103	6.029
El Salvador	42	25395	15.266	9230.231	604.643
Estonia	111	8885	102.865	8241.226	80.117
Eswatini	126	2909	196.686	4544.565	23.106
Ethiopia	5	265429	0.068	3600.819	53085.800
Finland	669	36230	127.181	6887.533	54.155
France	41299	621256	761.754	11459.094	15.043
Germany	35514	722405	433.312	8814.184	20.341
Greece	3870	76798	461.365	9155.294	19.844
Haiti	232	31462	47.679	6465.799	135.612
Hungary	539	61425	64.869	7392.572	113.961
Iceland	35	1892	200.959	10863.292	54.057
India	100875	5513476	148.830	8134.515	54.657
Indonesia	20085	1056765	186.437	9809.242	52.614
Iraq	160	91197	7.938	4527.312	570.338
Ireland	1758	26313	388.659	5817.613	14.968
Israel	2932	40283	437.598	6012.198	13.739
Italy	43793	477895	840.413	9171.083	10.913
Jamaica	298	12284	175.365	7223.975	41.194
Japan	825	1055426	8.931	11423.078	1278.994
Kenya	280	100001	11.708	4181.472	357.146
Latvia	42	12762	28.675	8713.226	303.857
Lithuania	138	18810	113.903	15536.782	136.403
Luxembourg	159	3454	234.104	5085.512	21.723

Table S3: Current and projected: COVID-19 deaths and YLL rates. *(continued)*

**Country**	**Deaths Current**	**Projected**	**YLL rates Current**	**Projected**	**Ratio of YLL Current over proj.**
Malawi	185	35425	20.559	3934.737	191.383
Malta	121	2914	401.552	9662.450	24.063
Mexico	122855	475425	2054.670	7951.175	3.870
Moldova	2825	19480	1052.198	7255.512	6.896
Nepal	360	103435	30.911	8881.387	287.319
Netherlands	11303	112170	666.451	6613.804	9.924
New Zealand	25	27680	6.476	7170.323	1107.200
Nicaragua	164	20112	52.275	6402.933	122.485
Nigeria	1163	391607	10.541	3549.960	336.779
Norway	353	32507	85.103	7836.956	92.088
Pakistan	1688	650513	15.724	6060.030	385.398
Panama	720	17275	366.026	8782.092	23.993
Peru	37835	131010	2319.726	8032.437	3.463
Philippines	9213	368084	173.797	6943.747	39.953
Poland	1132	239472	41.671	8815.485	211.548
Portugal	6191	74122	604.752	7240.540	11.973
Romania	842	121855	92.164	13337.987	144.721
Sierra Leone	72	16287	15.544	3511.335	225.895
Slovakia	878	31253	266.746	9495.005	35.596
Slovenia	2891	14051	1278.945	6216.000	4.860
South Africa	2413	198365	72.422	5954.112	82.214
South Korea	808	301352	19.466	7259.309	372.914
Spain	50900	321029	1219.862	7693.764	6.307
Suriname	54	2090	159.229	6162.769	38.704
Sweden	6553	66393	631.694	6400.128	10.132
Switzerland	7082	56606	780.948	6241.628	7.992
Taiwan	7	179828	0.688	17681.814	25689.714
Togo	66	16675	17.679	4466.559	252.652
Turkey	9799	337078	185.456	6379.428	34.399
Ukraine	17395	260783	606.804	9097.104	14.992
United Kingdom	73124	489828	1228.345	8228.183	6.699
Uruguay	90	20633	36.930	8466.471	229.256
USA	301671	2186315	1317.941	9551.578	7.247

Table S4: Gender imbalances: COVID-19 YLL rates, COVID-19 death counts, and ages-at-deaths.

	YLL per death		Mean age-at-death		Deaths	
**Country**	**Males**	**Females**	**Males**	**Females**	**Males**	**Females**
Afghanistan	18.619	21.436	60.069	58.205	1130	381
Argentina	14.653	14.713	70.670	75.887	19786	14973
Australia	9.790	8.273	82.951	87.372	440	470
Austria	10.160	8.893	80.768	85.516	1366	1107
Belgium	8.705	7.429	84.363	88.977	9652	10072
Bolivia	23.382	23.700	61.081	67.417	22	12
Brazil	17.188	18.547	68.832	71.476	106654	79753
Cameroon	15.855	1.880	62.841	95.160	13	5
Canada	10.368	9.814	81.290	84.954	4435	4754
Chad	15.299	20.160	65.068	59.574	70	22
Chile	16.434	16.156	71.597	75.609	8463	6314
Colombia	17.774	17.944	69.613	71.841	31736	17929
Croatia	13.313	11.316	73.670	80.468	208	192
Cuba	16.928	12.815	70.278	78.520	52	30
Cyprus	18.983	9.589	66.755	83.061	32	10
Czechia	10.869	10.224	77.418	81.335	6314	4944
Denmark	9.954	9.879	80.393	83.823	684	542
Dominican Republic	21.556	24.216	63.639	64.502	433	182
Ecuador	19.888	21.825	66.430	67.072	7025	3590
El Salvador	22.036	24.526	60.747	62.500	31	11
Estonia	11.169	12.816	77.073	79.721	42	69
Eswatini	14.960	21.446	61.056	58.674	72	54
Ethiopia	17.595	14.297	62.500	69.167	2	3
Finland	13.200	11.657	77.122	83.115	204	210
France	11.643	11.438	79.585	83.414	24268	17031
Germany	10.617	9.308	80.794	85.119	18573	16941
Greece	12.884	11.192	78.755	82.591	2314	1556
Hungary	11.441	10.973	75.255	80.525	264	275
Iceland	22.463	15.151	65.958	76.225	20	15
India	19.671	21.096	60.898	60.876	69887	30988
Iraq	17.927	23.070	62.146	58.572	110	50
Ireland	11.125	10.359	80.208	83.640	914	844
Israel	13.336	11.865	77.586	81.907	1670	1262
Italy	11.922	10.161	78.702	84.405	25257	18536
Japan	13.002	11.769	77.850	83.991	531	294
Kenya	20.669	25.100	58.510	56.019	210	70
Latvia	11.979	12.292	75.292	79.868	23	19
Lithuania	22.190	21.429	60.296	70.083	68	70
Luxembourg	9.918	7.938	81.985	87.455	82	77
Malawi	18.835	24.950	57.987	54.505	141	44
Malta	15.694	8.904	74.693	85.735	90	31
Mexico	20.744	21.932	62.993	64.463	77991	44864
Moldova	12.759	16.808	67.560	67.880	1457	1368
Nepal	23.248	28.083	56.092	52.662	258	102
Netherlands	10.395	9.469	80.520	84.222	6181	5122
Nigeria	17.811	20.577	57.915	55.668	869	294
Norway	13.735	11.192	76.635	83.367	189	164
Pakistan	19.949	21.929	60.743	58.865	1252	436
Panama	20.530	23.431	68.164	67.886	478	242

Table S4: Gender imbalances: COVID-19 YLL rates, COVID-19 death counts, and ages-at-deaths. *(continued)*

**Country**	**Males**	**Females**	**Males**	**Females**	**Males**	**Females**
Peru	19.163	20.778	65.959	67.618	25885	11950
Philippines	18.633	22.120	61.686	63.880	5554	3659
Poland	13.565	13.114	73.883	79.265	630	502
Portugal	10.161	9.244	80.822	85.255	3220	2971
Romania	18.842	23.124	63.897	63.426	500	342
Slovakia	15.910	16.557	69.309	73.267	452	426
Slovenia	9.597	8.374	80.879	85.929	1319	1572
Spain	11.440	10.214	80.039	84.971	27838	23062
Sweden	10.285	8.830	81.380	86.107	3563	2990
Switzerland	9.817	8.402	82.529	86.895	3811	3272
Taiwan	21.235	29.970	65.833	57.500	6	1
Togo	23.571	18.625	51.665	60.291	45	21
Turkey	15.267	15.589	70.199	74.033	6042	3756
Ukraine	13.132	16.531	67.968	69.026	9298	8097
United Kingdom	11.509	10.826	78.979	82.277	40213	32911
Uruguay	12.200	14.936	75.195	78.403	59	31
USA	14.802	13.499	74.381	79.228	163633	138038

Table S5: Ratio of equalizing to current deaths.

**Country**	**Equalizing proportion**	**Country**	**Equalizing proportion**
Afghanistan	0.409	Italy	0.593
Argentina	0.724	Japan	0.474
Australia	0.895	Kenya	0.401
Austria	0.690	Latvia	0.722
Belgium	0.866	Lithuania	0.844
Bolivia	0.552	Luxembourg	0.776
Brazil	0.779	Malawi	0.407
Cameroon	0.042	Malta	0.193
Canada	1.000	Mexico	0.582
Chad	0.407	Moldova	1.137
Chile	0.713	Nepal	0.402
Colombia	0.550	Netherlands	0.744
Croatia	0.729	Nigeria	0.402
Cuba	0.428	Norway	0.717
Cyprus	0.154	Pakistan	0.406
Czechia	0.715	Panama	0.579
Denmark	0.778	Peru	0.494
Dominican Republic	0.472	Philippines	0.789
Ecuador	0.561	Poland	0.724
El Salvador	0.356	Portugal	0.751
Estonia	1.687	Romania	0.793
Eswatini	1.041	Slovakia	0.929
Ethiopia	1.220	Slovenia	1.032
Finland	0.889	Spain	0.711
France	0.647	Sweden	0.728
Germany	0.782	Switzerland	0.723
Greece	0.563	Taiwan	0.232
Hungary	0.906	Togo	0.359
Iceland	0.505	Turkey	0.619
India	0.514	Ukraine	0.946
Iraq	0.598	United Kingdom	0.752
Ireland	0.848	Uruguay	0.601
Israel	0.666	USA	0.754

Table S6: Weighted Averages for Figure 1: Ratios of COVID YLL to Other Causes of Mortality

	Ratio	Deaths Weighted Average
1	Ratio COVID YLL to Flu (median year) YLL	0.793
2	Ratio COVID YLL to Flu (max year) YLL	0.557
3	Ratio COVID YLL to Transport YLL	0.572
4	Ratio COVID YLL to Heart disease YLL	0.096
5	Ratio COVID YLL to Excess mortality YLL	0.043

Table S7: Weighted Proportions for Figure 2: Proportion of total global YLL by age

	Age	Weighted Avg
1	Under 55	0.3017
2	55-75	0.4487
3	75+	0.2496

References

1. Martinez R, Soliz P, Caixeta R, and Ordunez P. Reflection on modern methods: years of life lost due to premature mortality-a versatile and comprehensive measure for monitoring non-communicable disease mortality. International Journal of Epidemiology 2019;48:1367–76.

2. Dicker D, Nguyen G, Abate D, et al. Global, regional, and national age-sex-specific mortality and life expectancy, 1950–2017: a systematic analysis for the Global Burden of Disease Study 2017. The Lancet 2018;392:1684–735.

3. Global Health Data Exchange — GHDx. 2020. URL: `http://ghdx.healthdata.org/` (visited on 06/01/2020).

4. Goldstein E, Viboud C, Charu V, and Lipsitch M. Improving the estimation of influenza-related mortality over a seasonal baseline. Epidemiology (Cambridge, Mass.) 2012;23:829–38.

5. Acosta E, Hallman SA, Dillon LY, et al. Determinants of Influenza Mortality Trends: Age-Period-Cohort Analysis of Influenza Mortality in the United States, 1959–2016. Demography 2019;56:1723–46.

6. Human Mortality Database. Available at www.mortality.org or www.humanmortality.de publisher: University of California, Berkeley (USA), and Max Planck Institute for Demographic Research (Germany). 2020. URL: `https://www.mortality.org/` (visited on 06/17/2020).

7. Walker PGT, Whittaker C, Watson OJ, et al. The impact of COVID-19 and strategies for mitigation and suppression in low- and middle-income countries. Science 2020;369:413–22.
